# A Systematic Review of Quantitative and Qualitative Results of Randomized Controlled Trials Assessing the Effect of Yoga in Adult Women With Post-traumatic Stress Disorder: What Is Known So Far

**DOI:** 10.7759/cureus.47765

**Published:** 2023-10-26

**Authors:** Marya Ali, Mrinal J P Oble, Shamsun Nahar Sonia, Sherie George, Srushti R Shahi, Zahra Ali, Abdelrahman Abaza, Aneeque Jamil, Sai Dheeraj Gutlapalli, Safeera Khan

**Affiliations:** 1 Psychiatry Clinical Research, Nishtar Medical University, Multan, PAK; 2 Psychiatry, California Institute of Behavioral Neurosciences & Psychology, Fairfield, USA; 3 Medicine, Kempegowda institute of Medical Sciences and Research Centre, Bengaluru, IND; 4 Internal Medicine, California Institute of Behavioral Neurosciences & Psychology, Fairfield, USA; 5 General Medicine, Pinderfields Hospital, Leeds, GBR; 6 Medicine, St. Martinus University Faculty of Medicine (SMUFOM), Willemstad, CUW; 7 Medicine, Bolan University of Medical and Health Sciences, Quetta, PAK; 8 Medicine, California Institute of Behavioral Neurosciences & Psychology, Fairfield, USA; 9 Pathology, California Institute of Behavioral Neurosciences & Psychology, Fairfield, USA; 10 Internal Medicine, Richmond University Medical Center Affiliated With Mount Sinai Health System and Icahn School of Medicine at Mount Sinai, Staten Island, USA; 11 Internal Medicine Clinical Research, California Institute of Behavioral Neurosciences & Psychology, Fairfield, USA

**Keywords:** yoga feasibility, yoga quantitative themes, hatha yoga, randomized clinical trial yoga, yoga rct, yoga in women, yoga for ptsd, women's mental health, yoga therapy, post traumatic stress disorder (ptsd)

## Abstract

Yoga has been increasingly practiced in recent years, with many of its practitioners being female. Adults may seek a complementary and alternative medicine (CAM) modality, such as yoga, to attempt to alleviate symptoms related to mental health conditions such as post-traumatic stress disorder (PTSD). Our goal for this systematic review was to collect and analyze all available data from quantitative and qualitative reports of randomized controlled trials (RCTs) conducted over the past 10 years on the impact of yoga on women diagnosed with PTSD. We included RCTs with the adult female population in which yoga was practiced for more than one week. We searched the databases PubMed, PubMed Central (PMC), and MEDLINE on June 11, 2022, Embase on June 12, 2022, and Science Direct on June 13, 2022, to find relevant articles. With the Cochrane RoB2 tool and Critical Appraisal Skills Programme (CASP) criteria, we checked for their quality, after which we selected 13 high-quality reports comprising seven original study designs and a total of 496 women. Of the 13 reports, nine evaluated effectiveness, four assessed feasibility, three explored acceptability, and four identified qualitative themes. We compared the results based on the assessed themes. Our results found yoga effective, feasible, acceptable, and a viable interoceptive pathway for emotional and personal growth. Limitations in our study include insufficient papers with large sample sizes and not including papers other than RCTs. With our research, we hope to present healthcare providers with research-based data on the effects of yoga so that they may better navigate its role in therapy as the trend of seeing more patients taking an interest in such alternative approaches rises.

## Introduction and background

While women experience fewer lifetime traumatic events than men, they have a two- to three-fold increased likelihood of developing post-traumatic stress disorder (PTSD) and tend to suffer from symptoms for longer [1-3]. The prevalence of PTSD during life is approximated to be nearly 10-12% for women and 5-6% for men [1].

Statistically, half of all women endure a minimum of one traumatic incident within their lifetime, with sexual assault being the most common cause of PTSD in both civilian and veteran women [1,4]. Civilian women experience almost two-thirds the frequency of lifetime PTSD prevalence (8.0%) as veteran women (13.4%) [5]. In contrast to the predominantly physical harm-induced PTSD of men, which includes combat-related trauma and motor vehicle collision, the nature of women’s traumatic experience is primarily interpersonal violence, including sexual assault, neglect or abuse in childhood, and domestic violence [1,6]. Their trauma is of higher impact and occurs at a much younger age, thus interfering with neurobiological and personality development [2]. Women rate higher PTSD subcluster scores and present more frequently with comorbid internalizing psychiatric disorders, including major depression, eating disorders, personality disorders, and anxiety disorders [7,8]. PTSD increases the risk of comorbid psychiatric conditions by 80%, leading to significant impairment in social, occupational, and physical functioning [3].

PTSD is classified as an anxiety disorder diagnosed by a set criterion from the Diagnostic and Statistical Manual of Mental Disorders-IV (DSM-IV) and occurs because of direct or indirect exposure to life-threatening trauma [3]. Symptoms must last more than one month and involve persistent re-experiencing of traumatic events, increased arousal to triggering cues, avoidance of related stimuli, negative adaptations in mental processing and affective states, hypervigilance, and a myriad of symptoms, including anxiety, nightmares, flashbacks, emotional detachment, negative mood; all which cause disturbed function in one or more areas of life [3]. Trauma-focused therapies using exposure techniques such as trauma-focused cognitive-behavioral therapy (CBT), cognitive processing therapy (CPT), eye movement desensitization and restructuring (EMDR), and prolonged exposure therapy (PET) are at the forefront of evidence-based treatments for PTSD. At the same time, the medication is typically an add-on therapy that can assist in symptomology but is not recommended as monotherapy due to low remission rates between 20% and 30% [6,9,10]. The long-term outcome for patients depends on the quality of social support received, one's ability to handle stress, substance abuse, and compliance with treatment [9]. Approximately 30% of patients recover with treatment, while 40% get better but exhibit less intense residual symptoms [9]. Individuals often seek alternate complementary and holistic approaches such as yoga for many reasons, including feasibility as shown by many clinical trials, accessibility, having another mental health issue, inability to tolerate the intense emotions confronted in conventional therapy, unresponsiveness to the conventional psychotherapies, and a philosophical orientation towards life and health [11-14].

Yoga, subclassified as a mind-body practice of complementary and alternative medicine (CAM), is a comprehensive mindfulness-based practice emphasizing attention to emotional and physical stimuli [15]. CAM is gaining popularity in the West as a tool for general well-being and prevention, with a national representative sample from a 2013 study showing that up to 40% of adults with PTSD reported using CAM in the past year to address mental and emotional health concerns [12]. It is easily accessible and can be done in a community setting or privately, and now virtually. Yoga, which unites the disciplines of somatic postures, respiratory exercises, and meditation is a historically dated practice for physical and mental wellness [16]. A recent survey data from Statista’s Global Consumer Survey from 2021 to 2022 shows that around 30% of the female population in the U.S. practices yoga, which is three times more than men [17]. According to a 2016 American study by the *Yoga Alliance and Yoga Journal*, over 70% of yoga practitioners are women, and 65% of yogis practice at home, with the most common motivator to start being flexibility [18].

While much has been published and compiled in systematic reviews about the efficacy of yoga in combat-induced PTSD patients, this relationship has not been compiled exclusively for PTSD in the adult female population who are at the forefront of yoga practitioners and thus are more likely to come to healthcare providers about queries regarding the effect of their yoga practice to their PTSD prognosis and treatment plan. This systematic review aims to fill this gap in the literature by compiling and presenting data from experimental studies published within the last 10 years.

## Review

Methods

The Preferred Reporting Items for Systematic Reviews and Meta-Analyses (PRISMA) principles were used to compile, oversee, and report the results of this systematic review [19].

Source Information and Search Strategy

An electronic literature search was performed using the following research databases: PubMed, PubMed Central (PMC), MEDLINE, Embase, and Science Direct. Each database was accessed on the following day: (1) PubMed, PMC, and MEDLINE on June 11, 2022; (2) Embase on June 12, 2022; (3) Science Direct on June 13, 2022. The dates of coverage for each database are as follows: (1) 2005 to present for PubMed PMC and MEDLINE; (2) 2002 to present for Embase; (3) 1976 to present in Science Direct. No filters and limits were used for any database. The search strategy is mentioned in Table 1.

**Table 1:** Summary of the Search Strategy PMC: PubMed Central, PTSD: Post-traumatic stress disorder, Mesh: Medical subject headings

Database		Search Strategy
PubMed, PMC, and MEDLINE	Medical Subject Headings (MeSH) Keywords	(( "Stress Disorders, Post-Traumatic/diagnosis"[Mesh] OR "Stress Disorders, Post-Traumatic/prevention and control"[Mesh] OR "Stress Disorders, Post-Traumatic/therapy"[Mesh] )) AND (( "Yoga/psychology"[Mesh] OR "Yoga/therapy"[Mesh] ))
PubMed, PMC, and MEDLINE	Keywords	‘PTSD AND Yoga’
Embase	Keywords	‘Post-traumatic stress disorder’ AND ‘yoga’ OR ‘PTSD’ and ‘yoga.’
Science Direct	Keywords	'PTSD AND yoga' OR 'post-traumatic stress disorder AND yoga.'

Inclusion and Exclusion Criteria

We included studies with an experimental design of a yoga intervention provided for more than one week to females between 18 and 65 years who were diagnosed with PTSD. Per our selection criteria, we excluded three conference abstracts lacking full text.

With the goal of this review in mind, the authors only included studies containing interventions that contained some form of yoga as a part of its mindfulness strategy and excluded studies that primarily focused on other CAM approaches such as meditation, dance, forest breathing, and Tai Chi. Studies evaluating a wide range of the effects of yoga were included to understand the known implications and effects thoroughly. The 15 reports that passed this stage of screening fulfilled each criterion. Two review authors independently screened the data, and a third reviewer was involved in case of conflict. No automation tool was used.

Our complete inclusion and exclusion criterion is mentioned in Table 2.

**Table 2:** Detailed Inclusion and Exclusion Criteria PTSD: Post-traumatic stress disorder, RCT: Randomized controlled trial

	Inclusion	Exclusion
Research Papers of Interest	Studies assessing PTSD-related symptoms	Studies assessing non-PTSD-related stress
Publication Date	Papers published in the last ten years (2012-2022)	
Literature	Published literature	Nonpublished literature, grey literature, conference abstracts, editorials, books, and documents.
Study Type and Design	RCT, pilot trials, RCT with interim results, randomized feasibility trials, secondary analysis of RCT, follow-up of an RCT, and qualitative reports of an RCT.	Non-randomized clinical trials, review, systematic review, meta-analysis, case reports, case series, observational studies.
Study Duration	Yoga longer than one week	
Study Setting	All settings	
RCT Sample Size	Papers with any sample size of RCT	
Population	Papers including women with PTSD due to all etiologies, papers including women from all races, papers including women from all countries, papers on veteran and civilian women	Papers including pediatric population, papers including geriatric population, papers including women who do not meet the criteria for a PTSD diagnosis
Sex of Population	Women	Men
Age of Population	Age 18-65	Younger than 18, older than 65
Species	Humans	Animals
Language	English	
Text Availability	Full text only	Conference abstracts of RCT

Results

Search Results

**Figure 1:**
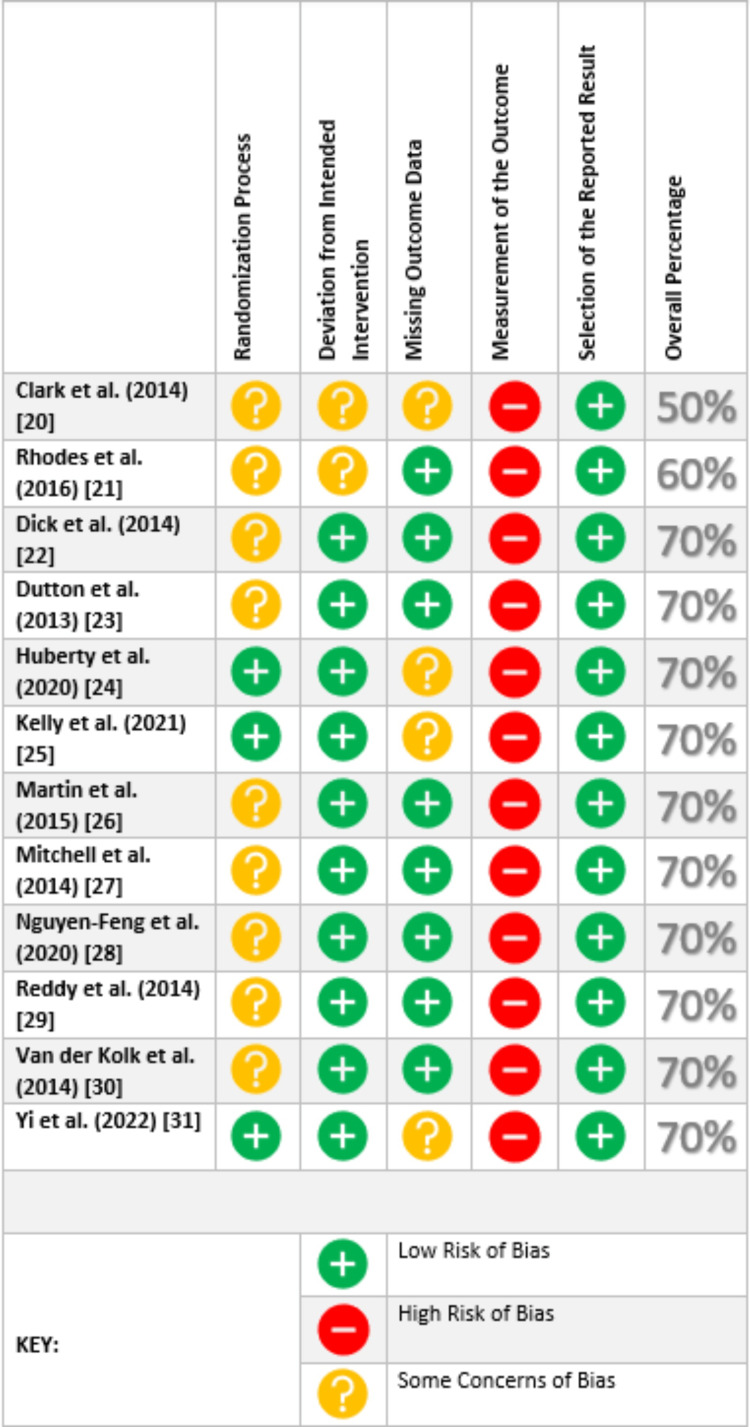
Cochrane Risk-of-bias Tool (RoB2) for Assessment of Randomized Controlled Trials (RCTs) The figure includes the quality assessment for [20-31].

Through our search strategy, we identified a total of 1675 articles which included: (1) 161 articles from PubMed, PMC, and MEDLINE; (2) 295 articles through Embase; (3) 1219 articles on Science Direct. No filters were applied. The articles were moved to an Excel sheet (Microsoft® Corp., Redmond, WA), where duplicates and non-English papers were removed manually, leaving us with 1465 records. One hundred forty-three papers remained after removing those with unrelated topics in their title, and these were further reduced to 32 after disqualification based on the abstract. Of these, the full texts of three conference abstracts were unavailable, leaving us with 29 papers that we screened for eligibility in accordance with our inclusion/exclusion criteria. The passing papers were 15 articles that were screened for quality appraisal. During the quality appraisal, two studies were excluded for not meeting the designated cut-off criteria, as shown in Figure 1 and Table 3 [20,21].

**Table 3:** Critical Appraisal Skills Program Y: Yes, C.T.: Can't Tell, N: No

Quality Assessment Criteria	Bermudez et al. (2013) [32]	Rhodes et al. (2015) [33]	West et al. (2017) [34]
Was there a clear statement of the aim of the research?	C.T.	Y	N
Was a qualitative methodology appropriate?	Y	Y	Y
Was the research design appropriate to address the aims of the research?	N	CT	Y
Was the recruitment strategy appropriate to the aims of the research?	Y	Y	Y
Was data collected in a way that addressed the research issue?	C.T.	C.T.	Y
Has the relationship between the researcher and participants been adequately considered?	N	CT	Y
Have ethical issues been taken into consideration?	Y	Y	Y
Was the data analysis sufficiently rigorous?	Y	Y	N
Is there a clear statement of findings?	Y	Y	Y
How valuable is the research?	Valuable	Somewhat	Valuable
Total score	7	8	8
Key: 1 point: Yes (Y) / Valuable. 0.5 points: Can’t Tell (C.T.) / Somewhat. 0 points: No (N) / Not Valuable.			

Finally, we were left with 13 high-quality papers for this systematic review, which consisted of six original RCTs, four secondary analyses of RCTs, and three qualitative studies.

Analysis of Study Quality/Bias

We critically rated 15 chosen articles for caliber using quality assessment tools of standard, 13 of which were of high quality and constituted in the review. The tools used are listed as follows: (1) for RCTs, the Cochrane Risk-of-Bias assessment tool (RoB2); (2) for qualitative studies, the Critical Appraisal Skills Program (CASP).

Six RCTs and four secondary analytic papers of original RCTs were assessed with RoB2, as shown in Figure 1. Blinding of participants is not always effective in psychological interventions, and all the studies comprise self-reported assessment tools given to participants in the intervention and control groups; therefore, a high risk of bias was always calculated in domain four, thus rating each study as a high-risk study as per the RoB2 tool calculation protocol. To deal with this issue, we modified the method for calculating the overall percentage of the studies as follows: each of the five domains was given an equal weight of 20%. A domain marked as ‘low bias’ attained a 20% score and a domain marked as ‘some concern for bias’ was given a 10% score. After computing the total score of the domains, studies with a minimum score of 70% were deemed high quality and included in this review. The details of the RoB2 results are mentioned in Figure 1.

To appraise qualitative research papers, all of which happened to be affiliated with a parent RCT appraised above, we used the CASP. Only the yoga intervention group was interviewed to gather qualitative data in all three papers. One article is a qualitative study carried out during a long-term follow-up of a previously completed RCT, one is a follow-up of interviews collected with a hermeneutic phenomenological approach, and the third is a report of the qualitative interview segment of an original RCT [32-34]. We included studies totaling a total score of seven or more. The specifics of the overall scores and quality of the individual studies are presented in Table 3.

PRISMA Flow Diagram

**Figure 2:**
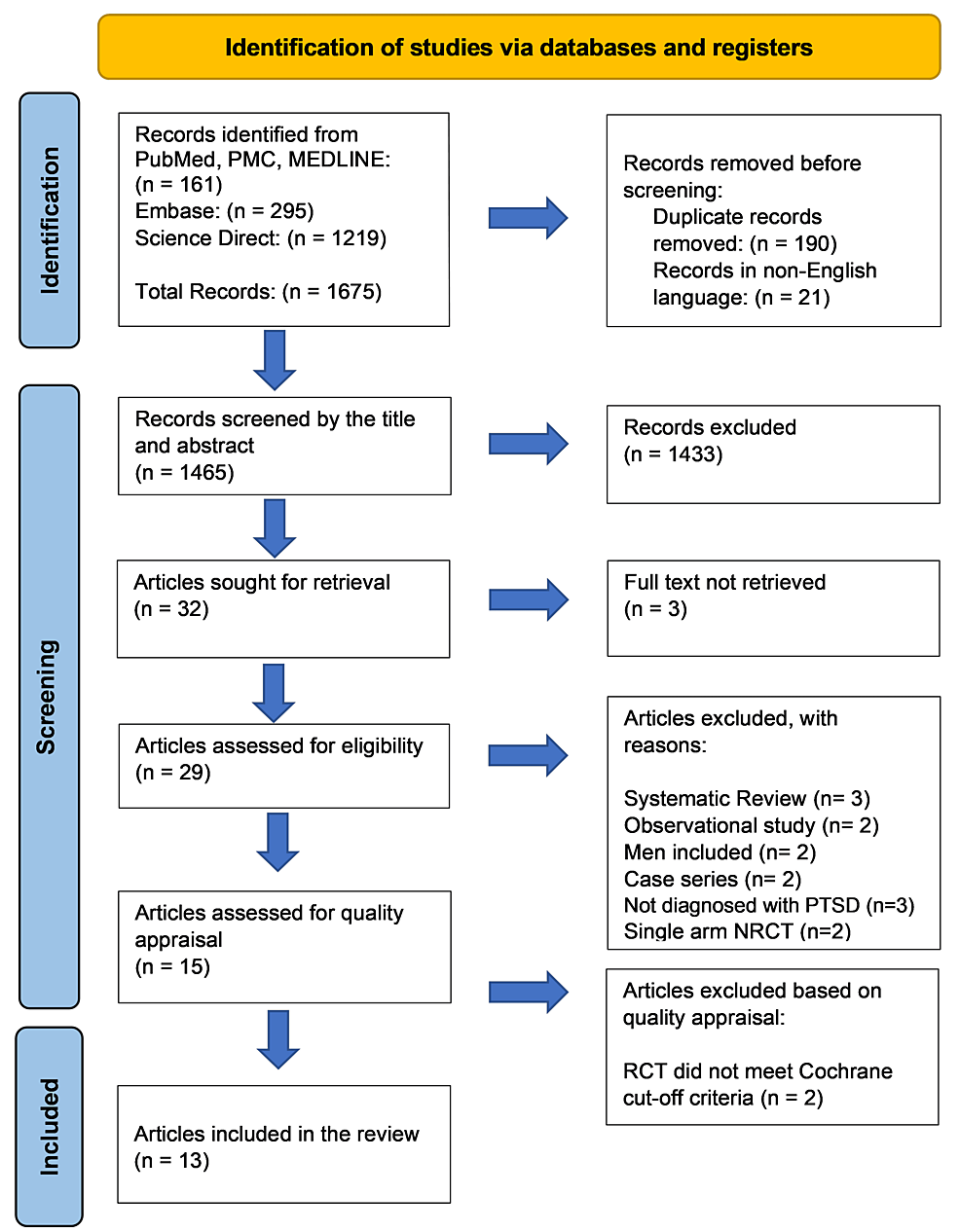
PRISMA Flow Diagram n: Number, PMC: PubMed Central, RCT: Randomized controlled trial, NRCT: Non-randomized clinical trial, PRISMA: Preferred Reporting Items for Systematic Reviews and Meta-Analyses

The PRISMA 2020-based diagram is outlined below in Figure 2 [19].

This review discusses information from seven unique studies comprising 496 female participants. Most studies use Trauma-Sensitive Hatha Yoga, while the control intervention varied as assessment only, stretch and tone, seminar, daily exchange, and CPT. Of the six RCTs in this review, two are pilot RCT studies, one is an online RCT, and one is an interim analysis of an RCT [23-25,27]. Four papers are secondary data analyses of an original RCT study [22,26,28,29]. The remaining studies include three qualitative reports of the results of RCTs [32-34]. Of the 13 reports, nine evaluated effectiveness, four assessed feasibility, three explored acceptability, and four identified qualitative themes that emerged surrounding the yoga practice. The studies assessed for quantitative findings such as effectiveness, feasibility, acceptability, and qualitative effects such as common cognitive themes experienced by the attending yogis, as seen in the summary Table 4.

**Table 4:** Summary of Studies and Reports AAQ-2: Acceptance and Action Questionnaire-II, A.A.: African American, A.I.: American Indian, AUDIT: Alcohol Use Disorder Identification Test, B: Baseline, BDI-2: Beck Depression Inventory, Second Edition, BREQ-2: Behavior Regulation in Exercise Questionnaire-2, C: Control, CAPS: Clinician-Administered PTSD Scale, CAPS-5: Clinician-Administered PTSD Scale for DSM-5, CES-D: Center for Epidemiologic Studies Depression Scale, CPT: Cognitive Processing Therapy, DASS-21: Depression Anxiety Stress Scales-21, DES: Dissociative Experiences Scale, DSM-5: Diagnostic and Statistical Manual of Mental Disorders, Fifth Edition, DTS: Davidson Trauma Scale, DUDIT: Drug Use Disorder Identification Test, ERQ: Emotional Regulation Questionnaire, F: Follow-up, GLTEQ: Godin Leisure-Time Exercise Questionnaire, I: Intervention, IASC: Inventory of Altered Self-Capacities, IASC-AD: Inventory of Altered Self Capacities, Affect Dysregulation, IASC-TR: Inventory of Altered Self Capacities, Tension Reduction Activities, IES-R: Impact of Event Scale-Revised, IPV: Intimate partner violence, LD: Low dose, M: Mid-treatment, MAAS: Mindfulness Attention Awareness Scale, MBSR: Mindfulness-Based Stress Reduction, MD: Moderate dose, MRI: Magnetic resonance imaging, MVA: Motor vehicle accident, P: Post-intervention, PC-PTSD: Primary Care Posttraumatic Stress Disorder Screen, PCL: Post-traumatic Stress Disorder Checklist, PCL-5: Posttraumatic Stress Disorder Checklist for DSM-5, PCL-C: PTSD Checklist-Civilian Version, PHQ-9: Patient Health Questionnaire-9, PSS-I: Posttraumatic Stress Disorder Symptom Scale - Interview, PTSD: Post-traumatic stress disorder, RCT: Randomized controlled trial, SEE: Self-Efficacy for Exercise, SF-12: 12-Item Short Form Health Survey, SCS: Self-Compassion Scale, STAI: State-Trait Anxiety Inventory, STC: Stretch and Tone Control, TCTSY: Trauma-Center Trauma-Sensitive Yoga, TSY: Trauma-Sensitive Yoga **These percentages are rough-rounded estimates.

Author (year)	Study Design	Population Assessed, Race/Ethnicity**	Number of Participants, Intervention (I), (Yoga Type and Study Setting), Control (C)	Setting of Study and Study Format	Study Frequency and Duration, and Time Points of Data Collection	Objective(s)	Data Collection Method for Primary Outcome	Secondary Outcomes/Parameters Assessed	Conclusion
Dick et al. (2014) [22]	RCT: Secondary data analysis of Mitchell et al.	Veteran and Civilian adult women. Race/ethnicity: A.A.: 37%, White: 53%, Other: 10%	See parent study (Mitchell et al.) I: Trauma-Sensitive Yoga (TSY), a Hatha-style yoga. C: Assessment only.	The meeting room at a medical center, Group sessions (Participants receiving additional standardized therapy not excluded)	Frequency/duration: see parent study (Mitchell et al.), Data collected at: B, P, F (1 month)	Explore the potential mechanisms by which either condition led to decreased PTSD in Mitchell et al.	A weekly questionnaire assessing themes related to Dialectical Behavior Therapy, Emotional Regulation Questionnaire (ERQ), Acceptance and Action Questionnaire-II (AAQ-2), Mindful Attention Awareness Scale (MAAS).	None	ERQ findings suggest that the suppression of one’s expression decreased with yoga. The reduction in PTSD symptoms in either group may be due to increased psychological flexibility measured by AAQ-2.
Dutton et al. (2013) [23]	RCT (Pilot study)	Low-income women with a history of chronic trauma, including intimate partner violence (IPV), who are predominantly African American (A.A). Race/ethnicity: A.A.: 67%, White: 23%, A.I.: 6%, Other: 4%	106; I: 53 (50%) C: 53 (50%). I: Mindfulness-Based Stress Reduction (MBSR) with Hatha Yoga. C: Not specified.	Community shelters for those residing there and community hospitals for those not living in shelters, Group sessions (Participants receiving additional standardized therapy not excluded)	Ten weeks duration (1.5-hour session per week) plus a single 5 hour retreat, Data collected at: B, M, P, F (3 months)	Feasibility and acceptability of yoga within the MBSR curriculum in low-income women who experienced intimate partner violence.	Interviews were conducted individually in a focus group format to gather data on feasibility and acceptability.	Qualitative themes of the interviews conducted.	MBSR is feasible and acceptable in low-income minority women who experienced IPV. Qualitative cognitive thematic findings include acceptance, awareness, belonging, compassion, reduced distress, self-efficacy, nonreactivity, and positive impact on daily life.
Huberty et al. (2020) [24]	RCT	Stillbirth-induced PTSD (grief within the last 24 months). Race/ethnicity: A.A.: 6%, White: 86%, A.I.: 1%, Other: 7%	90; I: (two groups with 30 women each) (66%) C: 30 (33%) I: Low-dose (LD) Hatha Yoga, Moderate-dose (MD) Hatha yoga. C: Stretch and Tone Control (STC).	Home-based (online delivery with premade prescribed videos), Solo sessions (Participants who were receiving additional standardized therapy were not excluded)	12 weeks duration; Low dose group: 60 minutes session/week, Moderate dose group: 150 minutes session/week, STC group: 60 minutes session/week. Data collected at: Feasibility and Demand at P (12 weeks), Acceptability at P and through a daily online survey, Preliminary efficacy measures at B, P (12 weeks), F (20 weeks)	Feasibility of yoga in this population using Bowen’s guidelines, which assesses acceptability, demand, and preliminary efficacy.	Impact of Event Scale-Revised (IES-R) for the assessment of PTSD manifestations.	Anxiety assessed with State-Trait Anxiety Inventory (STAI). Depression is assessed with Patient Health Questionnaire (PHQ-9). Perinatal Grief Scale to determine the amount of grief. Sleep quality is determined with Pittsburg Sleep Quality Index. Additional measurements with the 12-Item Short Form Health Survey (SF-12), Emotional Regulation Scale (ERQ), Mindful Attention Awareness Scale (MAAS), and Self-Compassion Scale (SCS).	Online yoga is effective and feasible. As for acceptability, less than half did not complete the study due to stress, availability, and emotional reasons. Despite this, those who dropped out enjoyed the experience. Most were satisfied and said social support would be of benefit.
Kelly et al. (2021) [25]	RCT	Veterans with military sexual trauma-induced PTSD. Race/ethnicity: A.A.: 91%, White: 1%, Other: 8%	104; I: 58 (56%) C: 46 (46%) I: Trauma-Center Trauma-Sensitive Yoga (TCTSY), a Hatha yoga style. C: Cognitive processing therapy (CPT).	Clinical site, Group sessions (Participants receiving additional standardized therapy not excluded)	TCTSY: 10 weeks; 60-minute session/week, CPT: 12 weeks; 90-minute session/week. Data collected at: B, M, two weeks after P, F (3 months)	Effectiveness relative to PTSD symptoms and severity.	Mini-International Neuropsychiatric Interview, Self-report with Post-traumatic Stress Disorder Checklist for DSM-5 (PCL-5), interview with Clinician-Administered PTSD Scale for DSM-5 (CAPS-5), psychological evaluation, and immunological measures.	None	TCTSY is effective for PTSD, with symptom improvement seen sooner than CPT. The effects are sustained afterward. Overall, no notable difference is seen in quality among TCTSY and CPT. Yoga was easier to access, afford, and more feasible for the veteran participants.
Martin et al. (2015) [26]	RCT: Secondary data analysis of Mitchell et al.	Veteran and Civilian adult women. Race/ethnicity: A.A.: 37%, White: 53%, Other: 10%	See parent study (Mitchell et al.) I: Trauma-Sensitive Yoga (TSY), a Hatha-style yoga. C: Assessment only.	The meeting room at a medical center, Group sessions (Participants receiving additional standardized therapy not excluded)	TSY: 12 weeks of 75-minute weekly sessions or six weeks of biweekly 75-minute sessions. Control: 12 weeks, meet once a week. Data collected at: B, P, F (1 month), GLTEQ: Weekly, P, F (1 month), SEE and BREQ-2: B, P, F (1 month)	Yoga’s effect on engaging in physical activity outside of yoga sessions. Yoga’s effect on motivation and self-efficacy for exercising.	Godin Leisure-Time Exercise Questionnaire (GLTEQ), A transtheoretical model measure of Self-Efficacy for Exercise (SEE), Behavior Regulation in Exercise Questionnaire-2 (BREQ-2).	None	A trend towards self-efficacy and motivation to increase leisurely exercise was seen in the yoga group only. One notable finding revealed a decline in exercise motivation driven by external directives, highlighting a shift towards internal motivation as the primary driving force.
Mitchell et al. (2014) [27]	RCT (Pilot)	Veteran and Civilian adult women. Race/ethnicity: A.A.: 37%, White: 53%, Other: 10%	38; I: 20 (53%) C: 18 (47%) I: Trauma-Sensitive Yoga (TSY), a Kripalu Yoga, which is a Hatha-style yoga. C: Assessment only.	A meeting room at a medical center, Group sessions (Participants receiving additional standardized therapy not excluded)	TSY: 12 weeks of 75-minute weekly sessions OR six weeks of biweekly 75-minute sessions. Control: 12 weeks; meet once a week. Data collected at: B, P, F (1 month)	Effectiveness of yoga on PTSD symptoms.	At baseline only: Primary Care PTSD Screen (PC-PTSD), Post-traumatic Stress Disorder Symptom Scale Interview (PSS-I), and Godin Leisure Time Exercise Questionnaire (GTLEQ). At B, P, and F: Post-traumatic Stress Disorder Checklist (PCL), Center for Epidemiologic Studies Depression Scale (CES-D), and State-Trait Anxiety Inventory (STAI). Weekly post-session questionnaire: Post-traumatic Stress Disorder Checklist-Civilian Version (PCL-C) and questions on mood, pain, and exercise.	Tolerance and feasibility of the intervention.	Yoga improved PTSD symptoms, but no compelling difference was detected between the two groups. PCL score significantly decreased in both groups, with a greater decrease in the yoga group. Decreases in all categories of the PCL-C score of both groups, including avoidance, hyperarousal, and re-experiencing. Yoga is tolerable and feasible.
Nguyen-Feng et al. (2020) [28]	RCT: Secondary data analysis of Van der Kolk et al.	Women with continual refractory PTSD secondary to interpersonal assaults that started in childhood. Race/ethnicity: White: 78%, Nonwhite: 22%	See parent study (Van der Kolk et al.) I: Trauma Center Trauma Sensitive Yoga (TCTSY), a type of Hatha Yoga. C: Women’s health education seminar in an interactive teaching style.	Unspecified setting, Group sessions (Administered as an adjuvant to ongoing psychotherapy and pharmacotherapy)	Ten weeks (1-hour session each week), Data collected at: B, M, P. The moderators were analyzed based on scores taken at B and P.	Extend the original study’s outcome by examining the non-demographic moderators of treatment efficacy for TCTSY. Provide clinicians with information on whom the yoga intervention worked best so that they may best prescribe the protocol with this awareness in mind.	Moderators assessed: cumulative interpersonal trauma, CAPS, Davidson Trauma Scale (DTS), Beck Depression Inventory 2nd edition (BDI-2), Dissociative Experiences Scale (DES), Inventory of Altered Self-Capacities (IASC) for emotional control problems (IASC-TR) and affect dysregulation (IASC-AD).	Examine the baseline characteristics that moderate the relationship between the intervention condition and PTSD severity outcomes, which includes examination of the scores from clinical assessment measures and the extent of exposure to adult interpersonal abuse.	TCTSY is more beneficial in those with less interpersonal trauma. This relationship is seen more prominently with CAPS, DTS, and emotional control scores.
Reddy et al. (2014) [29]	RCT: Secondary data analysis of Mitchell et al.	Veteran and civilian women with PTSD. Race/ethnicity: A.A.: 37%, White: 53%, Other: 10%	See parent study (Mitchell et al.) I: Trauma-Sensitive Yoga (TSY), a Hatha-style yoga. C: Assessment only.	The meeting room at a medical center, Group sessions (Participants receiving additional standardized therapy not excluded)	TSY: 12 weeks of 75-minute weekly session or six weeks of biweekly 75-minute sessions, Control: 12 weeks; meet once a week. Data collected at: B, P, F (1 month)	The aftermath of yoga in alcohol and drug use risks in PTSD diagnosed females.	Alcohol Use Disorder Identification Test, Drug Use Disorder Identification Test.	Treatment seeking questionnaire.	Yoga causes AUDIT and DUDIT scores to trend towards a decline.
Van der Kolk et al. (2014) [30]	RCT	Women with continual refractory PTSD secondary to interpersonal assaults that started in childhood. Race/ethnicity: White: 78%, Nonwhite: 22%	64; I: 32 (50%) C: 32 (50%) I: Trauma Center Trauma Sensitive Yoga (TCTSY), a type of Hatha Yoga. C: Women’s health education seminar in an interactive teaching style.	Unspecified setting, Group sessions (Administered as an adjuvant to ongoing psychotherapy and pharmacotherapy)	Ten weeks (1-hour session each week), Data collected at: B, M, P	Effect of yoga in women with treatment-resistant PTSD, given alongside standardized psychotherapy received for a minimum of 6 months, to assess if this complementary treatment would impact treatment-resistant symptoms.	CAPS (live and video recorded interviews) at B and P. Self-report measures: Inventory of Altered Self-Capacities (IASC), Davidson Trauma Scale, Dissociative Experiences Scale (DES).	Affect regulation and emotional control: assessed with IASC-AD and IASC-TR, respectively. Depression: assessed with Beck Depression Inventory-2 (BDI-2). Assess physiological response with heart rate variability. Brain activation: assessed with MRI.	The drop in PTSD scores for the yoga group was greater, with over half of the participants losing their PTSD status. DTS findings showed a reduction in PTSD in yogis but a relapse of symptoms in the control group after mid-treatment.
Yi et al. (2022) [31]	RCT	Women with PTSD caused by a motor vehicle accident (MVA) which happened at least three months ago. Race/ethnicity: No data available	94; I: 47 (50%) C: 47 (50%) I: Kripalu Yoga, a type of Hatha Yoga. C: Women exchanged their daily life experiences and played board games.	In a specific meeting room, Group sessions (Unspecified if adjuvant)	12 weeks (Six total sessions, 45-minute session every other week), Data collected at: B, P, F (3 months)	Effect of Yoga on psychological distress.	Impact of Event Scale-Revised (IES-R) for subjective self-perceived distress evaluation regarding their PTSD.	Anxiety, depression, and stress were assessed with the Depression Anxiety Stress Scale (DASS-21) to determine the extent of psychological distress.	Both groups showed improvements, with lower PTSD symptom scores in the yoga group. Reduction in emotional and mental anguish seen. Yoga is feasible and sustainable after MVA-induced PTSD.
Bermudez et al. (2013) [32]	Qualitative study: A follow-up of the intervention group from Dutton et al., an RCT.	Low-income women with a history of chronic trauma, including intimate partner violence. Race/ethnicity (at 9-month follow-up): A.A.: 90%, Other: 1%	At the 6-month follow-up: 53 women from Dutton et al. were followed. At the 9-month follow-up: 10 women from Dutton et al. were interviewed. I: Mindfulness-Based Stress Reduction (MBSR), which includes a yoga component. C: The control group was not followed for this follow-up.	Shelters that served only women, Group sessions (Participants receiving additional standardized therapy not excluded)	A 15-months total longitudinal study of the intervention group from Dutton et al. (an RCT). Data collected at: B M P F (3 months), F (9 months)	To offer considerations to providers who use mindfulness-based interventions in patients with a history of intimate partner violence, based on themes found after following adult women with intimate partner violence (IPV).	Conduct the study using the interpretive phenomenological analysis approach with interviews. Audiotape of four semi-structured interviews conducted and supervisor written notes on non-verbal observations.	None	The authors of the study advised that providers should know that women with a history of IPV benefit more from MBSR yoga sessions when attending it with other women with similar experiences. In the group setting, the instructor would only assist when needed, therefore the women achieved being able to discuss and process the trauma at their own rate in the group. It might be beneficial if the instructor recognizes the initial challenges of yoga, particularly when it comes to navigating heightened emotions, because this is to be expected as the women may pace the practice at their own processing rate.
Rhodes et al. (2015) [33]	Qualitative study: RCT (long-term follow-up of Intervention group from Van der Kolk et al.)	Women with chronic treatment-resistant PTSD secondary to interpersonal assaults that started in childhood. Race/ethnicity: A.A.: 10%, White: 77%, Other: 5%, Unreported: 8%	39; I: 39 women from Van der Kolk et al. C: none I: Trauma-Sensitive Yoga (TSY), a type of Hatha Yoga. C: none.	Unspecified setting, Group sessions (Administered as an adjuvant to ongoing psychotherapy and pharmacotherapy)	6-month period, Data collected at: F (follow-up timeframe varied per individual), the follow-up after the yoga varied based on which cohort group the individuals were in, ranging from 0.75 to 2.75 years.	Examine which experiences underlie yoga induced changes in functioning and quality of life addressed by previous literature, and how survivors experience yoga’s role within their coping and healing process over time.	Interviews in a semi-structured Hermeneutic phenomenological method, using audio recording and transcribed verbatim. The clinician-administered Stressful Life Experience Questionnaire.	None	The central concept of the qualitative findings lies within the scope of ‘claiming peaceful embodiment.’ This includes subthemes of living in the moment, improved understanding and responding to sensations, coping with stress and triggers, a sense of feeling grounded, improved self-care, intimacy, and hope. The study's findings uphold prior theories claiming that yoga assists with mental health through a calming effect and self-reflection opportunities.
West et al. (2017) [34]	Qualitative study: Qualitative interview of RCT yoga participants in Van der Kolk et al.	Women with chronic treatment-resistant PTSD secondary to interpersonal assaults that start in childhood. Race/ethnicity: A.A.: 10%, White: 74%, A.I.: 3%, Other: 13%	31 of the yoga intervention women. I: Trauma-Sensitive Yoga (TSY), a type of Hatha Yoga. C: no information given.	Unspecified setting, Group sessions (Administered as an adjuvant to ongoing psychotherapy and pharmacotherapy)	Ten weeks (1-hour session each week), Data collected at: P (after ten weeks of the TSY program)	First-hand data from those in the yoga group was taken to assess personal and symptomatic changes and their perception of the impact of TSY.	Semi-structured open-ended format interviews based on the PTSD and Organismic Valuing Theory. Interviews and field notes were interpreted using the qualitative descriptive methodology. Twenty-two of the women consented to be audiotaped and videotaped during the interview.	None	Five main intersecting themes were found: Gratitude and compassion, Relatedness, Acceptance, Centeredness, and Empowerment.

Data Extraction

The data derivation from qualified studies was executed independently by two review authors, and any discrepancies were resolved by involving a third reviewer. The reviewers assessed the studies based on pre-defined inclusion and exclusion criteria and evaluated their quality using agreed-upon appraisal tools. Only highly qualified studies were admitted in the final review, and no automation means were utilized.

Discussion

Yoga has been studied robustly for numerous physiological health-related ailments, and recently we are seeing an emerging of clinical trials being conducted for mental health conditions, with the first RCT which assessed the use of yoga in female-only PTSD participants being published in 2014 [27]. While the research conducted by Mitchell et al. was the first of its kind to solely study the correlation between PTSD prognosis and yoga, it was not until 2021 that an RCT compared yoga with an evidence-based psychotherapeutic intervention [25]. In many of the studies done, yoga was adjusted for the PTSD participants using a trauma-sensitive approach in which lower intensity moderations were made to the yoga technique to prevent the reemerging of trauma; therefore, creating a safe place for individuals and allowing them to choose if they want to proceed with the intervention as they navigate the emerging of any favorable and unfavorable bodily responses [23,25,27,30,31]. For example, the Trauma-Center Trauma-Sensitive Yoga (TCTSY) administered by Van der Kolk et al. uses invitational rather than command-based language. It encourages the noticing of sensations over achieving posture completion [30]. In all the studies, trauma-specific issues and disclosure of the trauma were not discussed in either group. Hatha Yoga is a foundation for Trauma-Sensitive Yoga (TSY) and was the assigned intervention in all six parent RCTs [23-25,27,30,31]. Hatha Yoga differs from other types of yoga as it comprises a meditative component and provides more of a restorative experience than a somatic exercise, is practiced at a slower rate, and focuses predominantly on breathing and perceived sensations of physical poses [35].

Effectiveness of Yoga

More in-depth details from the studies reporting the effectiveness of yoga are in Table 5.

**Table 5:** Findings of Studies Assessing Effectiveness AAQ-2: Acceptance and Action Questionnaire-II, AUDIT: Alcohol Use Disorder Identification Test, C: Control, CAPS: Clinician-Administered PTSD Scale, CAPS-5: Clinician-Administered PTSD Scale for DSM-5, CPT: Cognitive Processing Therapy, DASS-21: Depression Anxiety Stress Scales - 21 items, DTS: Davidson Trauma Scale, DUDIT: Drug Use Disorder Identification Test, ERQ: Emotional Regulation Questionnaire, I: Intervention, IES-R: Impact of Event Scale-Revised, IPV: Intimate partner violence, n: Sample size, PCL-5: Posttraumatic Stress Disorder Checklist for DSM-5, PCL-C: PTSD Checklist-Civilian Version, PTSD: Posttraumatic stress disorder, RCT: Randomized controlled trial, TCTSY: Trauma-Center Trauma-Sensitive Yoga, TSY: Trauma-Sensitive Yoga

Parent Study and Year of Publication	Population Demographics	Participants/PTSD Assessment Tool/Method	Effectiveness Measures Explored	Outcomes of Effectiveness	Conclusion	Outcomes Proposed by Secondary Analytic Studies	What the Study Adds to the Literature
Huberty et al. (2020) [24]	Women experiencing stillbirth grief in the last 24 months.	Total n = 90; I: Group 1: Online low-dose yoga (30), Group 2: Online medium-dose yoga (30), C: Stretch and tone routine (30). The Impact of Event Scale-Revised (IES-R) scores were measured at baseline, 12-week post-intervention, and 20-week follow-up. The study duration was 12 weeks. Low dose group: 60 minutes session/week, Moderate dose group: 120 minutes session/week, Stretch and tone routine: 60 minutes session/week.	Preliminary efficacy results were measured with the average minutes of weekly yoga performed. This was assessed by observing the low-dose and moderate-dose groups. IES-R scores were calculated to assess PTSD. A score of 33 or higher indicates positive PTSD diagnostic status. Secondary effectiveness outcomes measured include anxiety, depression, grief, mindfulness, self-compassion, and emotional regulation. Patients were assessed with self-questionnaires and an online software tracking tool.	The primary outcome (IES-R) continued to decrease, as seen with each data collection point, including at two weeks post-intervention. After this point, it began to rise, as was seen at the three-months post-intervention mark. Compared to the control, yoga caused a much greater drop in PTSD and depression scores, and this trend reversed with an increase in scores after 2-weeks postintervention. An average of a moderate to low dose session of 77 minutes of weekly yoga showed significant changes.	A possible dose-related effect of yoga can be seen, which proposes that every 10 minutes of yoga creates a potentially significant drop in the symptomatic findings (calculated score). These changes begin to reverse after prolonged abstinence from yoga practice.	Not Applicable	The first study to assess online yoga in the PTSD population. This study also examines the dose effect of yoga.
Kelly et al. (2021) [25]	Veteran-status women with military sexual trauma who have enrolled at the outpatient clinic anywhere between 2016 to 2020.	Total n = 104; I: TCTSY (58) C: CPT (46). CAPS-5 and PCL-5 scores, which are used to assess PTSD symptoms, were measured at different points in time: baseline, midpoint, and two weeks and three months after the intervention. Additionally, the four domains of PTSD, which include re-experiencing, avoidance, negative alterations, and hyperarousal, were measured. Ten weeks (1-hour weekly session) study duration.	A substantial change in the CAPS-5 score was determined as a minimum improvement of 10 points and a minimum of five points for PCL -5. Individual changes were assessed for each criterion. Completion of the condition was calculated by the percentage of attendees who appeared in at least 70% of the total sessions.	Both groups showed a substantial change in CAPS-5 and PCL-5 scores by the final data collection point at three months post-intervention. PTSD diagnosis scores dropped in both groups by the end of the study duration. The completion rate for TCTSY was significantly higher, reaching 60.3%, compared to CPT's rate of 34.8%. Follow-up shows sustained effects in both groups but slightly greater improvement for the CPT group.	For the yoga group, symptoms improved sooner at the midpoint mark, and twice as high individuals achieved attendance completion. This group also showed improvement in each CAPS-5 and PCL-5 criteria, especially in the avoidance category. No significant difference was seen in the results of both groups, and no intervention was deemed more efficient than the other; thus, yoga is just as effective as standardized therapy.	Not Applicable	The current study incorporates a gold-standard treatment as a control group. The gold standard is trauma-focused psychotherapy.
Mitchell et al. (2014) [27]	Veteran and civilian adult women.	Total n = 38; I: TSY (20) C: Assessment only (18). The PCL-C was measured at the baseline, postintervention, and 1-month follow-up. TSY: 12 weeks of weekly sessions or Six weeks of biweekly sessions; 75-minute-long sessions. Control: 12 weeks; once weekly.	PCL-C changes of 10-20 points implied a significant change in the outcome. Other effectiveness outcomes measured include depression and anxiety.	PCL-C score significantly decreased in both groups, with a greater decrease in the yoga group. An emerging relapse is seen at the 1-month follow-up. A decrease was seen in all categories of PCL-C scores of both groups, including avoidance, hyperarousal, and re-experiencing domains.	Yoga improved PTSD symptoms, but no compelling difference was noticed between the two groups. This pilot study shows yoga is tolerable and feasible.	Martin et al. (2015): a trend toward self-efficacy and motivation to increase leisurely exercise is seen only in the yoga group. A remarkable finding in the yoga group was a decrease in motivation by external directives. Reddy et al. (2014): There seems to be a trend toward a decline in AUDIT and DUDIT scores. Dick et al. (2014): ERQ findings suggest that suppressing one’s expression decreased with yoga. The reduction in PTSD symptoms in either group may be due to increased psychological flexibility, which was detected by AAQ-2.	First ever published RCT to evaluate the effect of yoga in the female PTSD population, a pilot study.
Van der Kolk et al. (2014) [30]	Women with long-standing refractory PTSD secondary to interpersonal violence, which began in childhood.	Total n = 64; I: TCTSY (32) C: women’s health seminar (32). CAPS interview and DTS were conducted at baseline, midpoint, and post-intervention. Ten weeks (60 minutes weekly session) duration.	A CAPS score below 45 indicates a loss of PTSD diagnosis. Secondary outcomes measured include emotional control, affect regulation, depression, and dissociation.	The CAPS score decreased in both groups, with a greater drop in the yoga group. 52% of yogis did not continue to meet the PTSD diagnostic benchmark at post-intervention versus 21% in the control group. DTS findings showed a reduction in PTSD in yogis but a relapse of symptoms in the control group after mid-treatment.	The drop in PTSD scores for the yoga group was greater, with over half of the participants losing their PTSD status by the last session.	Nguyen et al. (2020): TCTSY benefits those with less interpersonal trauma. This association is more noticeable among CAPS, DTS, and emotional control scores.	The first study of its kind to assess yoga’s effects in treatment-resistant patients with chronic IPV.
Yi et al. (2022) [31]	Women with PTSD due to experiencing a Motor Vehicle Accident a minimum of 3 months ago.	Total n = 94; I: Hatha Yoga (47) C: Daily exchange and board games (47). IES-R (Impact of Event Scale-Revised) scores were measured at baseline, postintervention, and the 3-month follow-up. 12 weeks (Six total sessions, 45-minute session every other week) study duration.	IES-R scores were looked at to see the changes in PTSD distress. Depression Anxiety and Stress Scale scores were assessed as well.	Both groups showed clinical improvement, with IES-R values being much lower for the yoga group than the control at the post-intervention follow-up. A similar trend was seen with DASS-21 items, except for unaffected stress scores. PTSD scores remained low during the 3-month follow-up.	Yoga decreased PTSD symptomatology, especially in the category of avoidance and intrusion. Yoga extended its effects also to decrease depression and anxiety scores.	Not Applicable	This study evaluates the effect of yoga in women with a history of motor vehicle accidents.

Kelly et al., published in 2021, is a unique RCT because it is the only study in which yoga was compared to a gold-standard control condition when scouting for its effectiveness in PTSD [25]. There was a steady decline of attendees in the CPT control group, just as expected based on previous literature which shows that gold-standard psychotherapy can cause an increased PTSD symptomology, thus a decline in attendance [25,36,37]. On the contrary, the attendance of the yoga group fell only slightly. Still, it remained steady until the completion of the last session at week 12 and had a 25% larger attendee retention rate [25]. No adverse events occurred in the yoga group, while two participants in CPT dropped out due to psychological distress [25]. Although attendee drop-out occurred in both conditions, the completion was nearly twice as high for the TCTSY group at 60.3% [25]. Both groups showed sufficient improvement in PTSD scores in all criteria, with the yoga group exhibiting sooner symptom improvement at the six-week midpoint mark, and its symptoms continued to improve from there onward, versus a delay in significant symptom improvement in the CPT study arm [25]. During the three-month post-treatment evaluation, the effect was sustained for both groups [25].

Yi et al., published in 2022, reported short-term benefits, including reduced avoidance symptoms and long-term improvements in thought intrusion [31]. The final scores for depression and anxiety were lower in the yoga group, which is important to consider as an additional benefit in our targeted population because of how common it is for these to be comorbid in PTSD individuals [31,38]. In 2020, Huberty et al. designed the first online RCT trial of yoga in the PTSD population, as was needed with the world moving online during the Coronavirus pandemic [24]. Despite the yoga being delivered online as individuals participated alone by following an instructor on premade videos, the findings suggest that individuals taking yoga had a greater drop in PTSD scores, perinatal grief, and depression scores than the control group, with the greatest difference seen between depression scores of the two conditions [24]. This brings to question the degree to which being in a group session impacts effectiveness. The study had two yoga groups based on the duration of yoga received [24]. The moderate dose group, which received 2.5 times the duration of yoga at 150 minutes per session, only showed a slightly greater drop in PTSD and anxiety scores than the low dose group, implying that doubling the duration does not double the effectiveness [24]. Because the moderate dose group had, on average, completed 77 of 150 minutes per week and showed such remarkable results, we can assume this amount of yoga is sufficient for significant changes. The drop in depression scores for both groups was similar [24]. In both yoga groups, PTSD and depression scores dropped significantly by the intervention completion time at week 12, but these were seen to project back up at the three-month follow-up [24].

Kelly et al.’s conducted study shows that the Clinician-Administered PTSD Scale (CAPS-5) score does not reverse as soon as stopping yoga sessions, as seen by the continuous decline at the two-week postintervention point [25]. The reversal is also seen at the three-month follow-up mark [25]. Mitchell et al.’s findings show a similar trajectory beginning at the one-month follow-up when the mean Post-traumatic Checklist (PCL) score rises by one to two points [27]. With these findings in mind, we can infer that the reversal of yoga effectiveness begins sometime between week two and one month of yoga-session abstinence, from whereon the trajectory continues to reverse. Conversely to that, the PTSD score was continually low at the three-month postintervention in Yi et al. [31]. Conducting a study that measures the trajectory of PTSD scores after three months or longer without yoga practice would provide insights into whether the scores return to their original levels, plateau, or follow a different pattern.

Van der Kolk et al., 2014, which used the clinician-rated CAPS tool as the primary assessment tool, concluded that the efficacy of yoga was greater than the control and more than twice the yogis lost their PTSD diagnosis compared to the control at the 10-week intervention completion point [30]. This is a remarkable finding considering the women in this study have chronic treatment-resistant PTSD [30]. The study also used the Davidson Trauma Scale (DTS), an additional PTSD measuring scale, which showed a continuous drop in the PTSD score of the yogis but a relapse in the score of the control group [30]. This is the only study where a follow-up after yoga abstinence was not done [30].

Nguyen et al., a secondary analysis published in 2020, further explored the findings of Van der Kolk et al. by analyzing who seemed to benefit most from the yoga intervention [28]. This was done by delineating the correlation between ‘adult relational trauma’ and ‘shifts in PTSD results’ over the passage of time for both groups [28]. Nguyen et al. accomplished this by assessing the non-demographic moderators between yoga and PTSD changes, which include cumulative interpersonal trauma and baseline mental health symptoms [28]. The analysis concluded that TCTSY was most efficacious in those with less interpersonal trauma, as shown by the changes seen in the moderator domains of clinician-assessed PTSD, self-reported PTSD, and Inventory of Altered Self-Capacities (IASC-TR) emotional control scores [28]. On the contrary, the domains of dissociation, depression, and IASC-AD affect dysregulation changes did not seem to be moderated by the traumatic experiences of the adult subjects [28]. Nguyen et al. stated that predicting the intervention efficacy in those with greater interpersonal traumas was tougher. Still, a dose responsiveness change may have to be considered for further probing [28].

Mitchell et al. was the first original study to perform an RCT of yoga in PTSD patients. Still, its power is <10% due to the low sample size, making any findings insufficient for declaring clinically significant outcomes [27]. It is, however, worth noting that almost all the yogis reported coping better than their control counterparts after the intervention [27]. The study showed yoga to lower PTSD scores in both conditions, which opened the gateway further to assess the yoga-PTSD relationship in more recent studies [27]. Three secondary data analyses were done using the data collected by Mitchell et al. to gather a greater understanding of the effects of yoga [22,26,29]. Martin et al. tested the applicability of the theories of self-determination, self-efficacy for exercise, and social cognition using yoga as a vehicle of physical activity [26]. A trend towards self-efficacy and motivation to increase leisurely exercise developed only in the yoga group, along with decreased motivation by external directives [26]. In Reddy et al., the change in Alcohol Use Disorder Identification Test (AUDIT) and Drug Use Disorder Identification Test (DUDIT) scores became apparent in the early stages of implementing the yoga protocol [29]. Among participants practicing yoga, the AUDIT and the DUDIT mean scores decreased; meanwhile, in the non-experimental arm, the AUDIT incremented, and DUDIT remained constant [29]. The study also found zero individuals categorized as high risk for drug or alcohol use after the intervention versus six in the control group [29]. These findings may be better understood with the Dialectical Behavior Therapy (DBT) themes of mindfulness, psychological flexibility, distress tolerance, effective action-taking, and emotional regulation, which Dick et al. analyzed secondarily from the Mitchel et al. parent study [22]. Data from that study revealed that the resulting psychological flexibility might contribute to PTSD symptom reduction in both groups, as seen by the Acceptance and Action Questionnaire (AAQ-2) tool [22]. Uniquely for the yoga group, a reduction in expressive suppression was noted [22]. These findings can help better understand the full scope of the potential use of yoga in the PTSD population.

Among the original parent RCTs, Van der Kolk et al. had the lowest yoga drop-out rate of 1.6%, and Huberty et al. had the largest drop-out rate, just below 50% [24,30]. All three groups in Huberty et al. had a similar drop-out rate which leads us to conclude the importance of group intervention and social support for treatment adherence [24]. The findings of Martin et al. may shed light on this, as it claims that the self-efficacy to practice solo yoga leisurely does not occur due to the lack of instructions and dexterity that comes with practice as complex as yoga, especially for beginners [26]. The decrease in PTSD scores seen in the control arms of every single included study raises questions about the role of social support and psychological determinants which may have aided these results; though, a greater reduction of scores in the yoga groups leads us also to consider the mind-body effects of yoga which are at play in the studies assessing effectiveness.

Most of the studies showed yoga to be of benefit, and the nonstandard controls also seemed to provide partial, if not equal, benefit to the PTSD participants. This inspires additional exploration of these conditions, which have positively impacted the participants. Yoga may assist with somatic prepping and mental aid when dealing with upcoming arousal or dysregulation associated with trauma-focused therapies; hence, adjuvant yoga may reduce the high rate of residual symptoms seen with such psychotherapies. It would be interesting to see how adjuvant yoga and psychotherapy will play out together in future studies.

Feasibility Accessibility and Demand of Yoga

Yi et al. found a weekly yoga strategy feasible in women with a history of a motor vehicle accident (MVA) [31]. Huberty et al. is a gateway study in determining feasibility, acceptability, and demand, as it is the first to create an online yoga protocol [24]. The formatted design constituted premade videos. Therefore, individuals lacked contact with other participants in the group and a real-time instructor [24]. Generally, those in the moderate dose group stated 150 minutes to be too much, while those in the lower dose group stated 60 minutes to be feasible for a yoga session [24]. Despite these claims, the completion rate in both groups was slightly over 50% [24]. Benchmarks for attendance and demand were not reached, with a little over half of the participants completing the study [24]. More than 90% of the participants suggested that a support group in future trials would help increase participation frequency, and 60% said they would suggest this yoga experience to others with a similar traumatic history [24]. The online premade course was easy, and more than half of the participants wanted to continue [24]. Common barriers to participation included not being in the mood, stress, and time management [24]. Mitchell et al., the first ever RCT of yoga done on PTSD participants, opened the doors to suggest yoga’s feasibility and demonstrated no adverse events with the practice [27].

Dutton et al. specifically looked at yoga feasibility for low-income women who were predominantly African American [23]. The completion rate was 70%, much higher than in other mindfulness-based yoga studies involving low-income minority women, indicating this to be acceptable and feasible for low-income women who lack access to conventional therapy [23]. Over half of the women expressed continual interest in participation after the study [23]. The population seemed to accept the practice, as most women practiced it informally outside the trial [23]. Yogis who practiced Mindfulness-Based Stress Reduction (MBSR) in Dutton et al. mentioned appreciating not having to talk about or focus on processing the trauma during the course [23]. Hatha Yoga does not require active trauma processing but only awareness of senses and is an interoceptive approach; thus, this may explain why individuals with trauma easily accept the approach.

Qualitative Themes Experienced by Attendees

The qualitative findings were found to extend beyond the yoga mat. Bermudez et al. showed that struggles to practice meditation lessened over time. By the midpoint, the whole group had grasped the concept of mindfulness and had begun to apply learned concepts to everyday situations [32]. Their struggles to practice meditation, such as visiting the traumatic experience without feeling judged, also seemed to decrease as they realized all group members had similar experiences [32]. For many, listening and responding to oneself compassionately was done for the first time on the yoga mat [33]. TSY skills (breathing, physical posture, sitting with emotions, evoking calm, gentle reminders) were behind qualitative findings such as better coping with stressors and triggers [33]. The psychological finding of less arousal may be compared to the clinically significant reduction in the CAPS and Impact of Event Scale-Revised (IES-R) avoidance category scores seen in Kelly et al. and Yi et al. [25,31]. Increased interoceptive awareness was initially positive for some and terrifying for others; some experienced body memories with certain poses, which led to non-visual flashbacks [33]. Some became more aware of their mind-body discomfort and pain, which they previously had not felt due to avoidance or dissociation [33]. West et al. found that remaining in difficult sensations and having greater tolerance for them benefits the individuals in psychotherapy. They proposed that this may decrease the drop-out rate in exposure-based therapy, and patients may increasingly engage in CPT [34].

Individuals in Dutton et al. detailed the benefits they perceived after their 10-week MBSR session; the most common benefits mentioned include mindfulness in other areas of daily life, a sense of belonging amongst the group format, compassion towards other’s suffering, less arousal, self-compassion, nonreactivity and controlling of one’s emotions, feeling greater self-efficacy, acceptance, tolerance of self and others, and being more aware of surroundings [23]. Bermudez et al. further followed up on yoga participants from Dutton et al.’s study nine months post-intervention to discover longitudinally present themes to provide clinicians with the most notable therapeutic benefits as they consider yoga for low-income women with chronic trauma [32]. An outlook of personal growth developed for oneself and others in a similar situation was felt and was expressed predominantly through setting professional goals. A novel approach was adopted in serving others, experiencing personal growth, and embracing opportunities for interpersonal growth [32]. Common areas of personal growth encompassed having mental serenity, awareness of self, emotional regulation as difficult feelings arise, and self-compassion [32]. Common areas of interpersonal growth include more easily pursuing friendships after years of low self-worth and isolation, communicating their needs as they began feeling heard more constructively and actively listening with openness, improved relationship quality, and feeling empowered with progressing in life after being able to sense and feel the pain and discomfort of emotions [32]. All these psychological effects grew with subsequent yoga sessions and were sustained nine months after the trial [32]. West et al. convey the qualitative aspect of the experience of yogis upon the completion of the Van der Kolk et al. study by using the PTSD and Organismic Valuing Theory, and it found five main postintervention themes, including gratitude and compassion, relatedness, acceptance, centeredness, and empowerment [34]. Rhodes et al. conducted a six-month follow-up of participants from Van der Kolk et al. to look at the qualitative cognitive themes using the hermeneutic phenomenon analysis [33]. The core theme discovered through the interviews with yoga participants was ‘claiming peaceful embodiment,’ which was then related to several interconnected themes [33]. Facilitators of the core theme and for continuing trial attendance include a gentle yoga approach, going at one's own pace, a choice-oriented approach, support from the instructor, and maintaining regular practice [33]. Barriers that hindered some participants from practicing yoga beyond this trial include cost per class, lack of internal motivation without a teacher’s assistance, fear of opening up, inability to access TSY, time restrictions, and fear that the improvement in symptoms would go away [33]. The body-focus approach gave them a mental, emotional, and physical present-moment body experience, and they felt less physically confined as a result of this new experience of the self [33]. Continuing an informal and incomplete version of the full yoga protocol outside the sessions was sufficient for coping with stress and trauma triggers [33].

**Figure 3:**
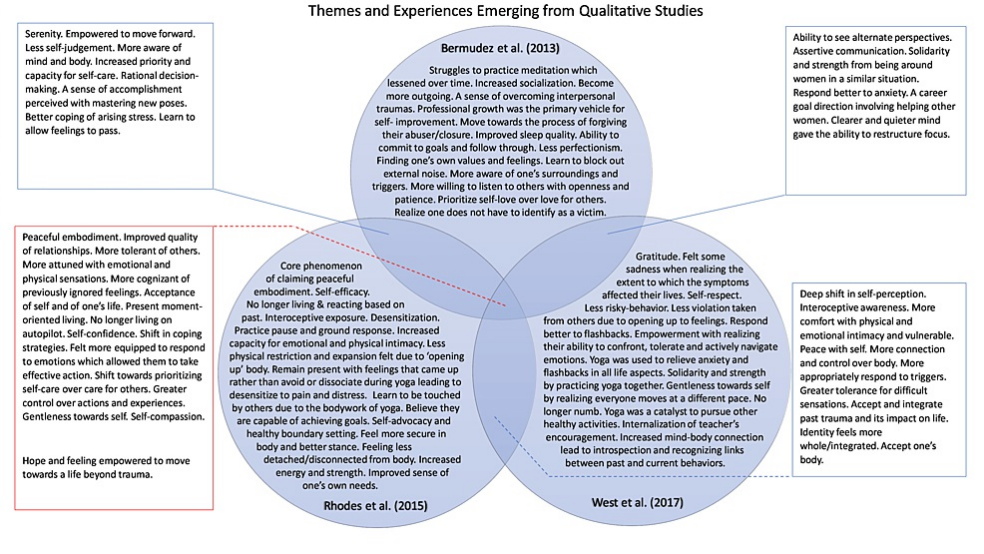
Themes and Experiences Emerging from Qualitative Studies Figure 3 was created by the first author using Microsoft PowerPoint (Sunnyvale, California). The Venn diagram depicted in Figure 3 seeks to compile the different elements of the themes, as identified in the qualitative studies included, which arose from the skills gained through Trauma-Sensitive Yoga (TSY) or Mindfulness-Based Stress Reduction (MBSR). The elements mentioned may have been experienced by one or more participants or even all, but not necessarily by every individual.

Commonalities and differences in qualitative study findings are represented in Figure 3.

Limitations

One prime limitation is our study lacks studies other than RCTs. We excluded some thought-provoking Non-Randomized Controlled Trials (NRCTs) and observational studies, which may have clarified additional gaps in knowledge. We did not find high-quality studies with large sample sizes, with the largest sample being 106 in Dutton et al. [23]. We did not find studies exploring the exact yoga dose and protocol components that led to effectiveness. It would be interesting to see the efficacy of yoga compared to a pharmacological treatment control group, considering that psychiatric medications can alter the cognitive processing of one’s mind [39]. This review includes women who developed PTSD from various events like stillbirth, intimate partner violence, MVA, treatment-resistant PTSD, and chronic trauma originating from childhood. We did not consider the PTSD incident, duration of onset, and severity when comparing the results. Based on the nature of gathering data for PTSD variables, the assessment tools in the studies were primarily self-reports, and few tools were clinician-administered; therefore, the findings have left room for considering the influence of psychosocial postulations and biases.

## Conclusions

We aimed to find the overall effects of yoga on women diagnosed with PTSD. Findings from the papers suggest yoga as a viable adjuvant or alternative PTSD treatment and a good substitute for those lacking access to or responding insufficiently to standard healthcare. Findings show yoga improves PTSD scores and those of related conditions, including depression, anxiety, emotional regulation, mindfulness, drug use, and alcohol use. Those with fewer adult interpersonal traumas may derive greater benefits from adjuvant yoga. Yoga seems just as effective as gold standard CPT. It is feasible and acceptable as it is less stigmatizing, less costly, group-focused for social support, and easily delivered in the community. Its inviting and self-navigating approach may be valuable for incorporating strategies for conscientiousness, self-regulation, and moving toward a greater quality of life. It provides profound benefits even in chronic treatment-resistant PTSD. We suggest that future studies have larger sample sizes so they may have greater power and potential to apply the findings to the general population. Moving on, more clarity will come in understanding the degree of effectiveness of yoga if trials control for additional variables such as the degree of PTSD at baseline, adjuvant therapies, PTSD onset and severity, and comorbid psychiatric conditions. This paper provides healthcare providers with the expectations and implications of prescribing yoga based on RCT findings, which is needed as society, especially women, has begun taking more interest in such CAM approaches.

No sponsors, financial assistance, or competing interests were involved in this systematic review.
